# P2Y_12_-Inhibitor Clopidogrel Promotes Collateral Artery Growth in a Murine Hindlimb Model of Arteriogenesis

**DOI:** 10.3390/biomedicines13112790

**Published:** 2025-11-16

**Authors:** Katharina Elbs, Lisa Bobrowski, Christoph Arnholdt, Matthias Kübler, Philipp Götz, Michael R. Rohrmoser, Daphne Merkus, Manuel Lasch, Elisabeth Deindl

**Affiliations:** 1 Institute of Surgical Research at the Walter-Brendel-Centre of Experimental Medicine, University Hospital, Ludwig-Maximilians-Universität München, 81377 Munich, Germany; katharina.elbs@med.uni-muenchen.de (K.E.); lisa.bobrowski@med.uni-muenchen.de (L.B.); christophjohannes.arnholdt@med.uni-heidelberg.de (C.A.); matthias.kuebler@med.uni-muenchen.de (M.K.); p.goetz@med.uni-muenchen.de (P.G.); michael.rohrmoser@med.uni-muenchen.de (M.R.R.); daphne.merkus@med.uni-muenchen.de (D.M.); manuel.lasch@med.uni-muenchen.de (M.L.); 2 Biomedical Center, Institute of Cardiovascular Physiology and Pathophysiology, Faculty of Medicine, Ludwig-Maximilians-Universität München, 82152 Planegg-Martinsried, Germany; 3 Department of Ophthalmology, University of Heidelberg, 69120 Heidelberg, Germany; 4 Deutsches Zentrum Immuntherapie (DZI) and Comprehensive Cancer Center Erlangen-EMN (CCC ER-EMN), Friedrich-Alexander-Universität Erlangen-Nürnberg (FAU), 91054 Erlangen, Germany; 5 Department of Oral- and Cranio-Maxillofacial Surgery, Friedrich-Alexander-Universität Erlangen-Nürnberg (FAU), 91054 Erlangen, Germany; 6 Department of Otorhinolaryngology, Head and Neck Surgery, Heidelberg University Hospital, 69120 Heidelberg, Germany; 7 Center for Cardiovascular Research (DZHK), Munich Heart Alliance (MHA), Partner Site Munich, 81377 Munich, Germany; 8 Department of Experimental Cardiology, Erasmus University Medical Center, 3015 Rotterdam, The Netherlands; 9 Department of Otorhinolaryngology, Head and Neck Surgery, University Hospital Munich, Ludwig Maximilians Universität München, 81377 Munich, Germany

**Keywords:** arteriogenesis, platelets, P2Y_12_-receptor inhibition, clopidogrel, vascular remodeling, cardiovascular occlusive diseases, coronary artery disease, peripheral artery disease, collateral artery growth

## Abstract

**Background/Objectives**: Clopidogrel is a P2Y_12_ receptor inhibitor commonly used as antiplatelet therapy for patients with cardiovascular occlusive diseases. However, its role in vascular remodeling remains poorly understood. Platelets orchestrate the sterile inflammation in arteriogenesis, an endogenous process to bypass an occluded artery. Therefore, we investigated the impact of P2Y_12_-inhibition by Clopidogrel on arteriogenesis. **Methods**: In this study, we utilized a well-established murine hindlimb model of arteriogenesis. To quantify the growth of collateral arteries, we employed laser-Doppler perfusion measurements and immunohistological analysis of growing compared to resting collateral arteries. Additional immunofluorescence and histological stains were conducted to assess immune cell recruitment and activation. Whole-blood flow cytometry was performed to analyze platelet–leukocyte interactions, and complete blood counts were obtained to quantify leukocyte and platelet numbers. **Results**: The findings of this study demonstrate that Clopidogrel promotes perfusion recovery following the induction of arteriogenesis compared to controls, attributed to elevated levels of proliferating vascular cells. Furthermore, compared to controls, Clopidogrel treatment significantly enhanced platelet-leukocyte interactions, increasing perivascular mast cell recruitment and degranulation, finally resulting in regenerative macrophage accumulation required for collateral artery growth. **Conclusions**: Clopidogrel treatment boosts arteriogenesis by enhancing the local regenerative inflammation relevant for vascular cell proliferation. Therefore, P2Y_12_ inhibition may represent a therapeutic option to effectively promote natural bypass growth in patients with cardiovascular occlusive diseases.

## 1. Introduction

The purinergic receptor P2Y subtype 12 (P2Y_12_) is a G protein-coupled receptor highly expressed on platelets, where it is activated by adenosine diphosphate (ADP) [1]. Its stimulation amplifies platelet responses by promoting aggregation and stabilizing thrombus formation [2]. Beyond platelets, P2Y_12_ receptors are expressed on certain vascular and immune cells, contributing to inflammatory signaling and vasodilation [3,4].

Pathological overactivation of P2Y_12_ is a key driver of arterial thrombosis, the underlying cause of life-threatening events such as myocardial infarction and ischemic stroke. In this context, excessive platelet activation promotes intravascular clot formation, thereby obstructing the blood flow and causing tissue ischemia [5]. Consequently, P2Y_12_ inhibition has become a cornerstone of antiplatelet therapy for patients with cardiovascular occlusive diseases. Antiplatelet agents, such as Clopidogrel, Prasugrel, and Ticagrelor, block ADP-induced platelet activation and reduce thrombus formation. Thereby, they have significantly improved cardiovascular outcomes, although their use is limited by the risk of bleeding complications [6,7].

Arteriogenesis describes the process of remodeling and enlargement of pre-existing arteriolar connections into functional collateral arteries by growth, thereby restoring perfusion after a major arterial occlusion [8]. Unlike angiogenesis, which is primarily hypoxia-driven, arteriogenesis is initiated by increased shear stress upon a hemodynamically relevant arterial stenosis. This activates the endothelium and initiates a cascade of regenerative inflammation [9,10], in which platelets play a crucial role. Following their activation via von Willebrand factor (VWF), platelets release growth factors and cytokines such as vascular endothelial growth factor (VEGF), platelet-derived growth factor (PDGF), and transforming growth factor ß (TGF-β) from α-granules, which promote vascular remodeling [11,12]. Moreover, platelet adhesion molecules such as P-selectin mediate platelet–leukocyte interactions that support immune cell recruitment, including mast cells and macrophages, that foster a sterile perivascular inflammation indispensable for collateral artery growth [13].

In the coagulation cascade, activated platelets release ADP, which enforces a positive feedback loop, further promoting platelet aggregation. ADP binds to the P2Y_12_ receptor, reduces vasodilator-stimulated phosphoprotein (VASP) phosphorylation via protein kinase G (PKG), thereby reducing the inhibition of GPIIb/IIIa-receptor and promoting platelet aggregation [14]. However, in the process of arteriogenesis, such excessive platelet activation leading to platelet aggregation could compromise the blood flow in the collateral arteries. Thus, endogenous counteracting mechanisms such as nitric oxide-mediated inhibition of glycoprotein IIb/IIIa (GPIIb/IIIa) are required. P2Y_12_ inhibitors can further support these endogenous mechanisms by enhancing VASP phosphorylation, suppressing GPIIb/IIIa activation, and ultimately preventing platelet aggregation [15].

Besides its well-described function in platelet biology, the P2Y_12_ inhibitor Clopidogrel has been proposed to exert pleiotropic effects, including increased coronary perfusion and anti-inflammatory signaling [16,17,18], both of which are possibly linked to the process of arteriogenesis. Therefore, in the present study, we evaluated the ability of the orally administered P2Y_12_ inhibitor Clopidogrel to improve arteriogenesis, as well as the underlying mechanisms.

## 2. Materials and Methods

### 2.1. Animal Model and Drug Treatment

All procedures involving animals complied with the European Directive 2010/63/EU for the protection of animals used in scientific research and received approval from the Bavarian Animal Care and Use Committee (approval IDs: Vet_02-17-99 and Vet_02-22-99). The animals were maintained under standard laboratory conditions with a 12 h light–dark cycle and had free access to food and water. The mice were randomly assigned to the respective treatment groups. Administration of the P2Y_12_-inhibitor Clopidogrel (Hycultec, Beutelsbach, Germany), based on a previously published study, began one day before femoral artery ligation (FAL). The compound was provided in drinking water at a daily dosage of 22.5 mg/kg body weight [19] to 8–12-week-old male SV129 wild-type mice (Charles River Laboratories, Sulzfeld, Germany). Mice were injected intraperitoneally each day with 100 μL bromodeoxyuridine (BrdU; 40.7 mM in phosphate-buffered saline, PBS, Merck, Darmstadt, Germany), starting immediately after FAL, to track proliferating vascular cells.

### 2.2. Femoral Artery Ligation (FAL)

A well-established murine hindlimb ligation model was used to induce collateral artery growth (arteriogenesis) [20]. For anesthesia, mice received a combination of fentanyl (0.05 mg/kg; CuraMED Pharma, Karlsruhe, Germany), midazolam (5 mg/kg; Ratiopharm GmbH, Ulm, Germany), and medetomidine (0.5 mg/kg; Pfister Pharma, Berlin, Germany) before the experiment. Following the standardized procedure of the murine hindlimb model, the superficial femoral artery was unilaterally tied off just distal to the origin of the profunda femoris artery. The opposite limb underwent a sham procedure, which served as an internal control. At the end of the experiment, blood was obtained by cardiac puncture into heparinized syringes. Subsequently, animals were sacrificed by cervical dislocation. Thereafter, hindlimb vasculature was perfused through an aortic catheter, first with 20 mL adenosine buffer [1% adenosine and 5% bovine serum albumin (BSA) in PBS; all reagents from Sigma-Aldrich, St. Louis, MO, USA], followed by 20 mL 3% paraformaldehyde (PFA) in PBS (Merck, Darmstadt, Germany) to preserve the tissue. Adductor muscles were then excised, immersed in 15% D(+) sucrose (AppliChem GmbH, Darmstadt, Germany) for 1 h, transferred to 30% sucrose overnight, and finally embedded in Tissue-Tek compound (Sakura Finetek Germany GmbH, Umkirch, Germany). The samples were stored at −80 °C and cut into 10-μm cryosections for histological examination.

### 2.3. Laser-Doppler Perfusion Imaging

**Figure 1:**
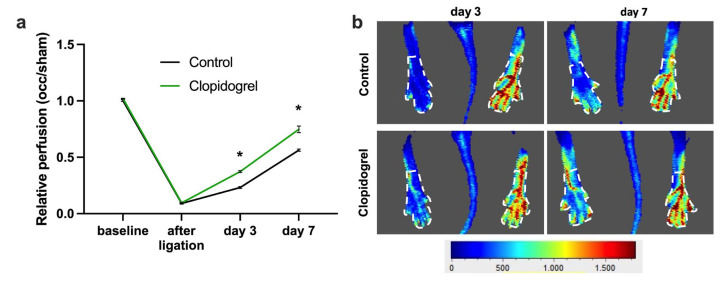
P2Y_12_ Inhibition Increases Perfusion Recovery after Femoral Artery Ligation. (**a**) The graph depicts the relative perfusion (calculated as the perfusion of the occluded side relative to the sham side of the same animal) in Clopidogrel-treated or control mice directly before (baseline), after, and on days 3 and 7 after femoral artery ligation (FAL). * *p* < 0.05 analyzed by two-way ANOVA (*n* = 6 animals). (**b**) Representative flux images of paws on day 3 and day 7 after FAL with the marked region of interest (white dotted lines) that was used for perfusion measurement. Color scale below the pictures: red = high perfusion, blue = low perfusion.

Recovery of hindlimb perfusion was monitored by laser-Doppler imaging (Moor LDI 5061 system, Moor Software v3.01; Moor Instruments, Remagen, Germany). The animals were anesthetized as described above and examined in a thermostatically regulated chamber to minimize variability caused by temperature. Scans were acquired prior to FAL, immediately after the procedure, and on postoperative days 3 and 7. For quantification, a standardized grid of 45 measurement points was applied to each paw (Figure 1). Blood flow values obtained from the ligated limb were expressed relative to those of the contralateral sham-operated limb, providing a measure of relative perfusion recovery.

### 2.4. Immunofluorescence and Histological Staining

Immunofluorescence staining was carried out to visualize proliferating vascular cells within collateral arteries as well as perivascular immune cell populations. The perivascular space was defined as the area immediately surrounding the collateral arteries, excluding adjacent skeletal muscle fibers.

For detection of proliferating cells, tissue sections of harvested adductor muscles on day 7 after FAL were subjected to DNA denaturation with 1 N HCl (Merck, Darmstadt, Germany) and permeabilization using 0.2% Triton X-100 (AppliChem, Darmstadt, Germany), followed by overnight incubation with a rat anti-mouse BrdU antibody (Abcam, Cambridge, UK, ab6326, 1:50). After rinsing with a wash buffer consisting of 0.5% BSA, 0.1% Tween in PBS (all from Merck, Darmstadt, Germany) a goat anti-rat Alexa Fluor 546–conjugated secondary antibody (Thermo Fisher Scientific, Waltham, MA, USA, A11081, 1:100) was applied. Endothelial cells were labeled with Alexa Fluor 647 conjugated anti-CD31 (BioLegend, San Diego, CA, USA, 102516, 1:100), and smooth muscle cells were identified using Alexa Fluor 488 conjugated anti–α-smooth muscle actin (ACTA2, Sigma-Aldrich, Darmstadt, Germany, F3777, 1:400). Nuclei were counterstained with DAPI (Thermo Fisher Scientific, Waltham, MA, USA, 62248; 1:1000 from 1 mg/mL stock).

Double labeling was performed with anti-CD68 (Abcam, Cambridge, UK, ab201844, 1:200) and anti-mannose receptor c type 1 (MRC1) to analyze perivascular macrophages to distinguish M1-like from M2-like polarized macrophage subsets. Sections from adductor muscles harvested on days 3 and 7 after FAL were then incubated with a rabbit anti-human MRC1 primary antibody (Abcam, Cambridge, UK, ab64693, 1:200), followed by Alexa Fluor 546-conjugated anti-rabbit IgG secondary antibody (Thermo Fisher Scientific, Waltham, MA, USA, A10040, 1:200).

Mast cell recruitment and degranulation were assessed by Giemsa staining of adductor muscle tissue sections harvested 24 h and 3 days after FAL following a standard protocol [21].

All stained samples were imaged at 40× magnification using a Leica DM6 B epifluorescence microscope system (Leica Microsystems, Wetzlar, Germany). Image processing and quantitative analysis were performed with Fiji (ImageJ, Version 1.54p) software [22]. To determine the diameters of collateral arteries, the inner luminal collateral circumference was measured and used for the calculation of the diameters using the formula: inner luminal diameter = circumference/π. For all immunofluorescence and histological stains, we selected two collateral arteries from each animal and examined three sections from each artery. We used the average value from each animal for statistical analysis to ensure that each animal was counted as one biological replicate.

### 2.5. Flow-Cytometry

Flow cytometry was employed to characterize circulating immune cell subsets and to quantify the formation of platelet–leukocyte aggregates (PLAs) after induction of arteriogenesis. Heparinized whole blood was collected by cardiac puncture 24 h after FAL after anesthetizing the mice as described above. To remove erythrocytes, samples were treated with a 1:10 dilution of the lysing solution (10× concentrate, BD Biosciences, Franklin Lakes, NJ, USA, 349202) prepared in distilled water. The remaining leukocytes and platelets were subjected to surface marker staining with fluorophore-conjugated antibodies. Specifically, CD11b-PE was used as a pan-leukocyte marker (BioLegend, San Diego, CA, USA, 101208, 1:300), CD115-BV421 identified monocytes (BioLegend, San Diego, CA, USA, 135513, 1:300), CD41-FITC labeled platelets (BioLegend, San Diego, CA, USA, 133903, 1:400), and Ly-6G/GR-1-APC detected neutrophils (BioLegend, San Diego, CA, USA, 108412, 1:400). A fixable viability dye (Invitrogen, Waltham, MA, USA, EF780, 1:1000) was included to exclude non-viable cells and ensure accurate quantification.

Acquisition was performed on a BD LSRFortessa™ flow cytometer (Becton, Dickinson and Company, Franklin Lakes, NJ, USA). Gating on leukocyte subpopulations, especially neutrophils and monocytes, and identifying CD41 co-expression enabled the detection of platelet–leukocyte aggregates.

### 2.6. Differential Blood Analysis

To analyze the different cell subtypes in animals that underwent femoral artery ligation, we performed a differential blood analysis using the ProCyte DX (Idexx, Westbrook, ME, USA) with mouse-specific settings. For this, blood was collected via cardiac puncture from anesthetized mice as described above 24 h after FAL directly before neck dissection.

### 2.7. Statistical Analyses

All statistical analyses were carried out using GraphPad Prism 10 (GraphPad Software, La Jolla, CA, USA). Unless otherwise stated, results are presented as mean values accompanied by the standard error of the mean (SEM), which estimates the variability of the group mean. A *p*-value of less than 0.05 was considered statistically significant. To ensure transparency and reproducibility, the corresponding figure legends specify the statistical test used for each dataset. The normality of the data was assessed using the Shapiro–Wilk test before performing further statistical analyses.

Sample sizes were determined a priori with G*Power software (Version 3.1.9.2), based on expected effect sizes derived from preliminary data or previous publications. This approach was chosen to achieve adequate statistical power and thereby reduce the likelihood of false negatives.

## 3. Results

### 3.1. P2Y_12_ Inhibition by Clopidogrel Boosts Perfusion Recovery After Femoral Artery Ligation

To induce collateral artery growth, we performed unilateral femoral artery ligation (FAL) while conducting a sham surgery on the contralateral side as an internal control. Hindlimb perfusion after FAL was assessed by in vivo laser-Doppler imaging. The results of our study show that the perfusion after FAL decreased to approximately 10% compared to the sham side and then recovered on days 3 and 7 after FAL in all experimental groups. Mice that received Clopidogrel treatment starting the day before FAL exhibited a significantly improved perfusion recovery compared to control animals on day 3 and day 7 after FAL. Of note, Clopidogrel treatment did not affect the relative perfusion before and directly after FAL (Figure 1).

### 3.2. Clopidogrel Treatment Enhances Vascular Cell Proliferation In Vivo

**Figure 2:**
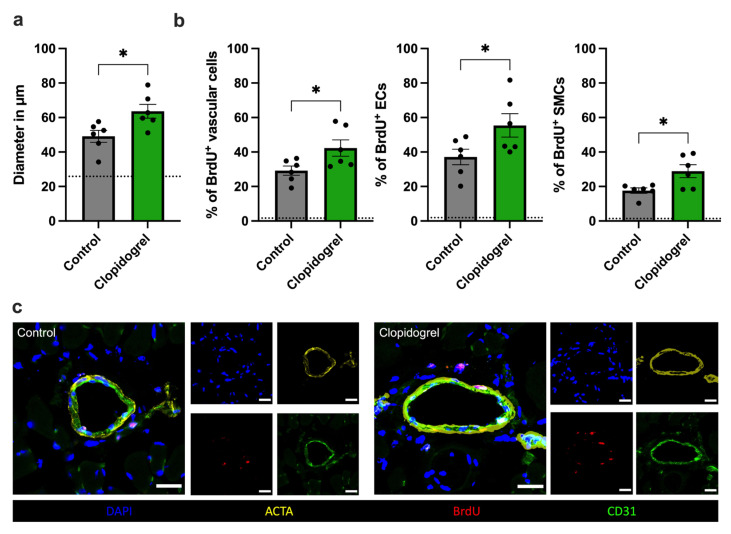
Clopidogrel Increases Collateral Artery Diameter by Promoting Vascular Cell Proliferation Upon Induction of Arteriogenesis. (**a**) The bar graph shows the inner luminal diameter of collateral arteries 7 days after induction of arteriogenesis by femoral artery ligation (FAL) in Clopidogrel-treated or control mice. (**b**) The bar graphs show the percentage of bromodeoxyuridine-positive (BrdU^+^) vascular cells per collateral artery, specified as BrdU^+^ endothelial cells (EC) relative to all EC and BrdU^+^ smooth muscle cells (SMC) relative to all SMC, in Clopidogrel-treated and control mice (*n* = 6 animals, analysis of 2 collateral arteries in 3 sections per animal). The dotted line in (**a**,**b**) represents the mean values of the sham side. Statistical analyses were performed using the unpaired two-tailed *t*-test. * *p* < 0.05. (**c**) Representative immunofluorescence images of collateral arteries from control and Clopidogrel-treated mice 7 days after FAL. The large images display merged versions of the smaller single-channel images. DAPI (blue) was employed for nuclei-staining, anti-smooth muscle actin alpha 2 (ACTA2, yellow) to mark SMCs, CD31 (green) to label ECs, and BrdU (red) as a proliferation marker. The overlap of CD31 and BrdU appears pink in the merged images. Scale bar = 25 μm.

To determine whether the improved hindlimb perfusion recovery on days 3 and 7 after FAL in Clopidogrel-treated animals was a result of arteriogenesis rather than other influencing factors, such as vasodilation, we conducted immunofluorescence staining of the harvested adductor muscles harboring collateral arteries on day 7 post-FAL. Here, we examined BrdU incorporation to identify proliferating cells of all vascular cells following the induction of arteriogenesis. The results of this assay demonstrate that P2Y_12_ inhibition by Clopidogrel significantly enhanced collateral artery diameters and, more importantly, increased vascular cell proliferation compared to the control group. When differentiating for the vascular cell subpopulations, we found significantly enhanced proliferation of endothelial and vascular smooth muscle cells in Clopidogrel-treated mice compared to their controls (Figure 2).

### 3.3. Clopidogrel Promotes Platelet-Leukocyte-Aggregate Formation During Arteriogenesis

Clopidogrel is most well-known for its role as an inhibitor of the P2Y_12_ receptor on platelets. In this study, we aimed to investigate its effect on platelet activation during arteriogenesis. Previous research has highlighted the vital role of platelet activation and its interaction with leukocytes in arteriogenesis [13,23].

**Figure 3:**
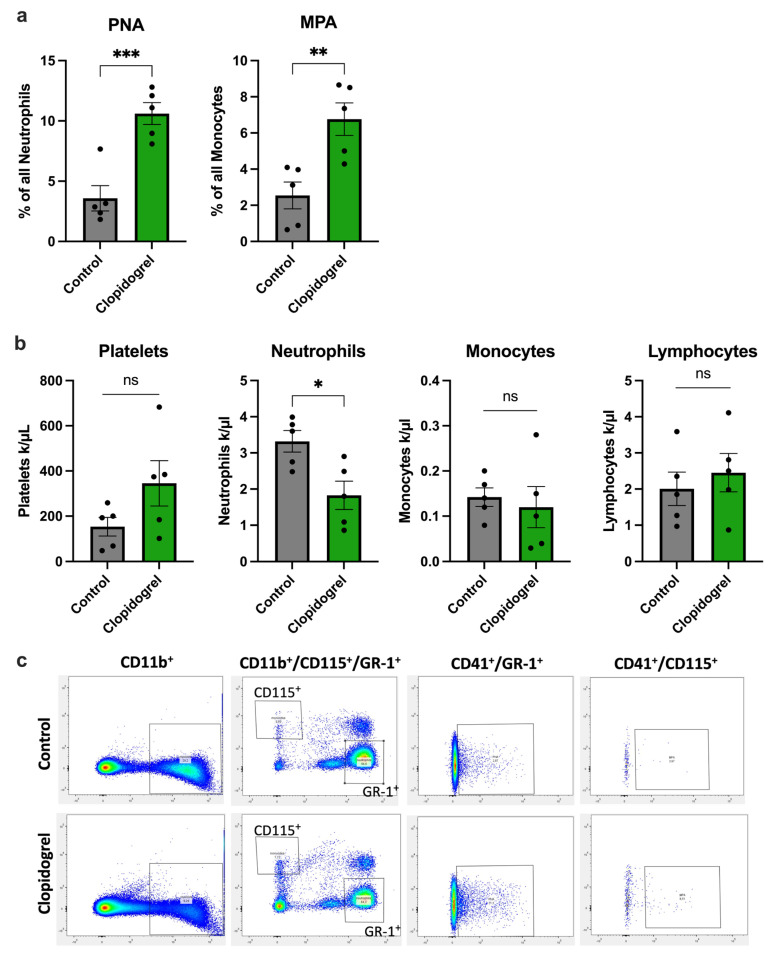
Clopidogrel Treatment is Associated with Enhanced Platelet-Leukocyte Aggregate Formation. The bar graphs demonstrate (**a**) the percentage of platelet-neutrophil-aggregates (PNA) of all neutrophils, and the percentage of monocyte-platelet-aggregates (MPA) of all monocytes in blood collected 24 h after induction of arteriogenesis in Clopidogrel-treated or control mice, analyzed by flow-cytometry. (**b**) The bar graphs depict the number of platelets, neutrophils, monocytes, and lymphocytes in whole blood collected 24 h after induction of arteriogenesis in Clopidogrel-treated or control mice, analyzed via differential blood count. (**a**,**b**) Statistical analyses were performed using the unpaired *t*-test (*n* = 5 animals). ns no significance. * *p* < 0.05, ** *p* < 0.01, *** *p* < 0.001. (**c**) Representative dot plots from flow cytometry analyses of whole blood are shown for control and Clopidogrel-treated mice 24 h after the induction of arteriogenesis. These analyses detect leukocytes (CD11b^+^), followed by the differentiation of monocytes (CD115^+^) and neutrophils (GR-1^+^) within these leukocytes. Additionally, in this cohort, the presence of double-positive cells is examined to identify PNA (CD41^+^/GR-1^+^) and MPA (CD41^+^/CD115^+^).

In this study, we conducted flow cytometry analyses on whole blood samples collected 24 h after the induction of arteriogenesis by FAL. Here, dual staining of leukocytes with the platelet marker CD41 demonstrated that Clopidogrel treatment significantly increased the levels of platelet-neutrophil aggregates and monocyte-platelet aggregates (Figure 3a) compared to the control group. Importantly, the total number of monocytes and lymphocytes, as evaluated by differential blood count, did not show a significant difference after Clopidogrel treatment. The platelet count tended to be higher in Clopidogrel-treated mice compared to the controls, although this failed to reach statistical significance. However, there was a significant reduction in neutrophil counts in Clopidogrel-treated mice compared to the controls (Figure 3b).

### 3.4. Enhanced Perivascular Mast Cell Recruitment and Degranulation upon Clopidogrel Treatment in Arteriogenesis

**Figure 4:**
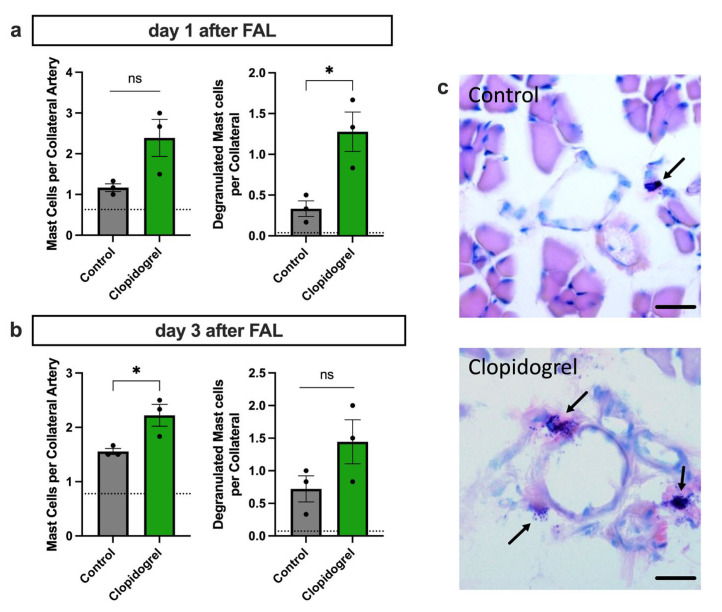
Clopidogrel Treatment Boosts Mast Cell Recruitment and Degranulation in the Perivascular Space of Growing Collaterals. The bar graphs show the number of recruited (left) and degranulated (right) mast cells around growing collaterals in Clopidogrel-treated or control mice on day 1 (**a**) and day 3 (**b**) after induction of arteriogenesis by femoral artery ligation (FAL) as evaluated by counting the numbers of perivascular mast cells on Giemsa-stained tissue samples. The dotted line in (**a**,**b**) represents the mean values of the sham side. Statistical analyses were performed using the unpaired *t*-test (*n* = 3 animals, analysis of 2 collateral arteries in 3 separate sections per animal). ns no significance. * *p* < 0.05. (**c**) Representative Giemsa stains of mast cells (marked with arrows) around growing collateral arteries of Clopidogrel-treated or control mice 24 h after FAL. Scale bar = 25 μm.

By releasing stromal-derived factor 1 alpha (SDF-1α), platelets contribute to the recruitment of different immune cells, such as mast cells, to the perivascular space of growing collaterals [13,24]. The interaction of platelets with neutrophils leads to the production of reactive oxygen species (ROS) in neutrophils, which in turn stimulates mast cell degranulation. To evaluate whether this process is influenced by Clopidogrel treatment, we performed Giemsa stains on collateral arteries harvested 24 h or 3 days after the induction of arteriogenesis. Our study demonstrated a clear increase in the recruitment of perivascular mast cells and a significant rise in mast cell degranulation on day 1 after FAL in Clopidogrel-treated animals compared to the control group (Figure 4a,c). By day 3 post-FAL, the number of recruited mast cells in the perivascular space of the growing collaterals was significantly elevated in the Clopidogrel-treated group compared to controls, underscoring the drug’s impact on mast cell dynamics during arteriogenesis (Figure 4b).

### 3.5. Clopidogrel Boosts Regenerative Inflammation Around Growing Collateral Arteries

Mast cell degranulation contributes to an inflammatory environment around arteries, which promotes the recruitment of monocytes and their maturation into macrophages [13]. To investigate macrophage recruitment, we performed immunofluorescence staining on tissue samples collected on day 3 and day 7 following FAL. Our goal was to analyze the potential early (day 3) and late (day 7) responses to Clopidogrel treatment during arteriogenesis.

**Figure 5:**
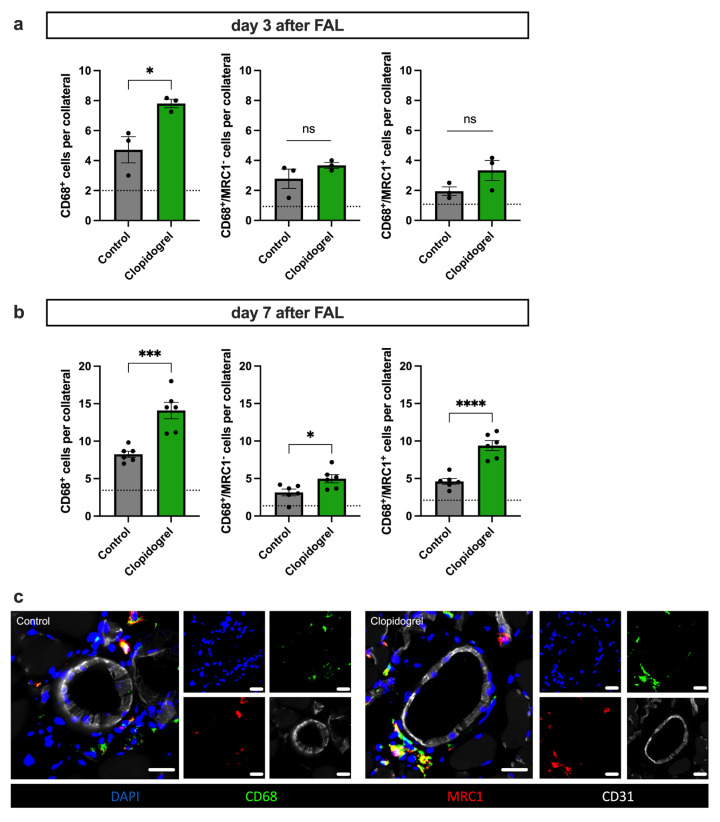
Clopidogrel Treatment Enhances Perivascular Macrophage Recruitment in Arteriogenesis. (**a**,**b**) The bar graphs show the total amount of macrophages (CD68^+^ cells, left), M1-like polarized macrophages (CD68^+^/mannose receptor type 1 (MRC1)^-^ cells, middle), and M2-like polarized macrophages (CD68^+^/MRC1^+^ cells, right) per collateral artery in Clopidogrel-treated or control mice 3 days (**a**) and 7 days (**b**) after induction of arteriogenesis by femoral artery ligation (FAL). The dotted line represents the mean values of the sham side. Statistical analyses were performed using the unpaired two-tailed *t*-test ((**a**): *n* = 3 animals, (**b**): *n* = 6 animals; analysis of 2 collateral arteries in 3 separate sections per animal), ns no significance, * *p* < 0.05, *** *p* < 0.001, **** *p* < 0.0001. (**c**) Representative immunofluorescence staining of collateral arteries of Clopidogrel-treated or control animals on day 7 after FAL. The large images display merged versions of the smaller single-channel images. DAPI (blue) was used for nuclei-staining, CD68 (green) to mark macrophages, CD31 (white) to label endothelial cells, and MRC1 (red) as a marker for M2-like macrophage polarization (the overlap of CD68 and MRC1 appears as yellow in the merged images). Scale bar = 25 μm.

Our data show that Clopidogrel treatment significantly enhanced the recruitment of perivascular macrophages to the perivascular space of growing collateral arteries on day 3 after FAL. When examining the different subsets of macrophages, no significant difference was observed between M1-like polarized macrophages (mannose receptor c type 1 (MRC1^−^)) and M2-like polarized macrophages (MRC1^+^) at this time point (Figure 5a). By day 7 after FAL, Clopidogrel-treated mice still had significantly more macrophages recruited to the perivascular space of growing collaterals compared to the control group. However, at this time point, there was a significant increase in M1-like and M2-like polarized macrophages, whereby the shift towards the M2-like polarization was much more pronounced in the Clopidogrel-treated group (Figure 5b,c).

## 4. Discussion

Cardiovascular occlusive diseases remain the leading cause of death worldwide [25]. Therefore, novel non-invasive therapeutic strategies are urgently needed to improve patient outcomes. One promising concept is the stimulation of arteriogenesis, the growth of natural arterial bypasses that restore blood flow upon an arterial occlusion or stenosis. Despite its potential, no clinically feasible pharmacological approach is currently available to effectively promote arteriogenesis. Platelets have emerged as key modulators not only in thrombosis, but also in vascular remodeling processes [26]. In particular, inhibition of the platelet P2Y_12_ receptor has been demonstrated to improve outcomes following percutaneous coronary intervention [27] and to attenuate vascular inflammation [18]. Based on these findings, we aimed to investigate the role of P2Y_12_ receptor blockade by Clopidogrel in arteriogenesis.

In this study, we demonstrate that Clopidogrel treatment significantly enhanced perfusion recovery following femoral artery ligation in mice compared to controls. Immunofluorescence staining of collateral arteries following the induction of arteriogenesis revealed a significant increase in proliferating endothelial and vascular smooth muscle cells in Clopidogrel-treated mice compared to the control group, suggesting true collateral growth rather than vasodilation. Our findings contrast with those of previous studies from the early 2000s, which examined the influence of Clopidogrel in much lower doses on arteriogenesis and found no significant impact, either positive or negative [28,29]. This discrepancy is likely explained by differences in dosing and species-specific vascular responses. In the present study, we selected a dosing regimen frequently used in mouse studies [19,30] that has been shown to effectively inhibit the P2Y_12_-receptor. Finally, it is possible that advancements in perfusion and histological techniques may enable more sensitive detection of treatment effects.

Mechanistically, P2Y_12_ inhibition reduces platelet activation, thereby limiting the release of vasoconstrictive [31] and pro-inflammatory mediators [32] and creating a more permissive environment for vascular remodeling. Additionally, reduced interactions between platelets and endothelial cells may decrease excessive inflammatory signaling that could impair collateral artery growth. However, the P2Y_12_ receptor is also found on vascular and immune cells [3,4]. To determine which mechanism contributes to the beneficial effects of Clopidogrel on the growth of collateral arteries, we examined various steps in the process of arteriogenesis [33].

After the induction of arteriogenesis by femoral artery ligation, the laminar shear stress in the collateral arteries increases. This rise in laminar shear stress causes activated endothelial cells to release VWF [34]. As a result, platelets are stimulated to express P-selectin on their surface. This enhances the interaction between platelets and leukocytes, a crucial step in initiating the inflammatory cascade that promotes positive remodeling of the collateral arteries [13]. Here, we show that Clopidogrel treatment significantly upregulated platelet-neutrophil and monocyte-platelet aggregate formation, which contrasts with the previous observation that Clopidogrel can inhibit platelet-leukocyte interactions [35,36]. However, our findings indicate that P2Y_12_-inhibition may not directly suppress these interactions, as evidenced by the observed increase in platelet–leukocyte aggregates. This may suggest a compensatory increase rather than a direct stimulatory effect of Clopidogrel on PLA formation.

Importantly, Clopidogrel significantly reduced absolute neutrophil counts in mice. This phenomenon has already been described in patients and might represent an additional beneficial effect of Clopidogrel treatment in patients with cardiovascular occlusive diseases [37]. The activation of the complement system stimulates neutrophils, which in turn adhere to and potentially damage the endothelium [38,39]. Thus, the reduction in neutrophil counts by Clopidogrel treatment may protect the vessels from neutrophil-mediated damage. Moreover, the reduction in absolute neutrophil count and the observed tendency towards an increase in platelet counts might cause the compensatory rise in platelet-leukocyte-aggregate formation in Clopidogrel-treated animals.

As platelet activation and PLA-formation are prerequisites for mast cell degranulation [13], we performed Giemsa stains to evaluate mast cell responses in Clopidogrel-treated mice. The results from this analysis demonstrate that Clopidogrel significantly enhanced mast cell degranulation compared to controls 24 h after induction of arteriogenesis by FAL. Furthermore, Clopidogrel-treated animals exhibited significantly more recruited mast cells to the perivascular space of growing collateral arteries 3 days after FAL. Activation of the P2Y_12_ receptor by ADP has been reported to reduce the bioavailability of ROS required for mast cell degranulation, whereas inhibiting the P2Y_12_-receptor with Clopidogrel diminished this effect in vivo [40]. These data support our findings, highlighting a novel role for Clopidogrel in promoting ROS-dependent mast cell degranulation during arteriogenesis.

Mast cell degranulation creates a permissive environment for monocyte recruitment from the circulation into the perivascular space of growing collateral arteries [13,41]. Once recruited, monocytes differentiate into macrophages that secrete pro-arteriogenic cytokines, growth factors, and matrix-remodeling enzymes. These macrophages are crucial for collateral artery growth, as they stimulate the proliferation of vascular endothelial and smooth muscle cells required for functional arteriogenesis [42,43]. Here, we show that Clopidogrel significantly increased total macrophage counts on days 3 and 7 after FAL. Additionally, we noted a shift towards a higher M2-like regenerative macrophage polarization in the perivascular space of growing collaterals in Clopidogrel-treated mice. Matrix metalloproteases released from perivascular M2-like polarized macrophages contribute critically to outward collateral artery remodeling [44,45]. The seen shift towards a more regenerative macrophage polarization upon Clopidogrel treatment indicates an anti-inflammatory property of Clopidogrel in the later phases of arteriogenesis. Consistent with our findings, Clopidogrel treatment has been reported to negatively affect the P2Y_12_-activation-induced macrophage-to-myofibroblast transition [46]. The potential of Clopidogrel to exert anti-inflammatory effects by downregulating inflammatory markers, such as monocyte chemoattractant protein 1 (MCP-1) and interleukin 8 (IL-8), was highlighted in a different study [47]. We have recently demonstrated that MCP-1 is associated with M1-like macrophage polarization during arteriogenesis [48]. Accordingly, reduced levels of MCP-1 following Clopidogrel treatment may explain the shift from M1-like towards increased M2-like polarized macrophages in the perivascular space of growing collateral arteries, as observed in our study.

Clopidogrel treatment has been shown to reduce levels of plasminogen activator inhibitor-1 (PAI-1), a major inhibitor of urokinase-type plasminogen activator (uPA) [49]. Since uPA activity is required for extracellular matrix degradation and facilitates the infiltration of leukocytes into the perivascular space [20,50], lowering PAI-1 levels may further enhance the uPA-mediated recruitment of leukocytes that drive collateral artery growth [18]. This mechanism provides an additional layer of explanation for the pro-arteriogenic effects of P2Y_12_ inhibition, suggesting that Clopidogrel not only modulates platelet and mast cell responses, but also promotes leukocyte recruitment to the perivascular space of growing collateral arteries through regulation of the fibrinolytic system.

Since Clopidogrel is widely prescribed for patients with cardiovascular occlusive diseases, translation of our findings into clinical practice is feasible, but also subjected to limitations. First, it would be of great interest to quantify collateral artery diameters in patients treated with Clopidogrel compared to those who are not. Additionally, a heterogeneity in the response to Clopidogrel caused by genetic polymorphisms [51], drug–drug interactions [52], or diabetes [53] has been described. This raises the question of whether laboratory testing and personalized treatment can optimize the use of Clopidogrel in individual patients [54]. Moreover, the use of Clopidogrel is limited by the risk of bleeding complications [55]. Due to this risk, Clopidogrel is not suitable for long-term management, particularly in elderly patients with cardiovascular occlusive diseases who may already have heightened susceptibility to bleeding due to age-related factors or comorbidities. As a consequence, healthcare providers must carefully consider the benefits and risks of Clopidogrel for this vulnerable population. Given the bleeding risk associated with long-term systemic Clopidogrel treatment [55], local perivascular drug delivery may enhance efficacy while reducing adverse effects, as demonstrated for other cardiovascular drugs in previous studies [56]. However, its requirement for invasive wrap implantation currently limits its practicality.

Currently, acetylsalicylic acid is most commonly used as an antiplatelet monotherapy for secondary prevention in patients with cardiovascular occlusive diseases [57]. Recent studies have demonstrated that P2Y_12_-inhibitor monotherapy is potentially superior to acetylsalicylic acid monotherapy for long-term secondary prevention in patients with coronary artery disease [27,58]. Our findings may provide an additive benefit for this emerging trend by showing that Clopidogrel promotes arteriogenesis, providing a mechanistic rationale for its potential superiority over acetylsalicylic acid in secondary prevention.

In conclusion, this study demonstrates the beneficial effect of high-dose Clopidogrel treatment on collateral artery growth in mice. Here, P2Y_12_-inhibition led to elevated numbers of platelet-leukocyte-aggregates associated with an increased number of activated mast cells in the perivascular space of growing collaterals. This resulted in the accumulation of regenerative macrophages surrounding the collaterals, fostering vascular cell proliferation, and hence arteriogenesis. Thus, we suggest Clopidogrel as a ready-to-test drug for the treatment of patients with cardiovascular occlusive diseases.

## Data Availability

The original data presented in this study are included in the article material. Further inquiries can be directed to Lisa Bobrowski (lisa.bobrowski@med.uni-muenchen.de).

## Abbreviations

The following abbreviations are used in this manuscript:

ACTA2	Anti-alpha smooth muscle actin
ADP	Adenosine diphosphate
BrdU	Bromodeoxyuridine
BSA	Bovine serum albumin
FAL	Femoral artery ligation
GPIIb/IIIa	Glycoprotein IIb/IIIa integrin receptor
MCP-1	Monocyte chemoattractant protein 1
MPA	Monocyte-platelet-aggregate
MRC1	Mannose receptor C type 1
P2Y12	Purinergic receptor P2Y subtype 12
PAI-1	Plasminogen activator inhibitor-1
PBS	Phosphate-buffered saline
PDGF	Platelet derived growth factor
PFA	Paraformaldehyde
PKG	Protein kinase G
PLA	Platelet-leukocyte-aggregate
PNA	Platelet-neutrophil-aggregate
ROS	Reactive oxygen species
SDF-1α	Stromal-derived factor 1 alpha
SMC	Smooth muscle cell
TGF-ß	Transforming growth factor ß
uPA	Urokinase-type plasminogen activator
VASP	Vasodilator-stimulated phosphoprotein
VEGF	Vascular endothelial growth factor
VWF	Von Willebrand factor
